# Prospective memory in prodromal Alzheimer's disease: Real world relevance and correlations with cortical thickness and hippocampal subfield volumes

**DOI:** 10.1016/j.nicl.2020.102226

**Published:** 2020-02-22

**Authors:** Volkan Nurdal, Alfie Wearn, Michael Knight, Risto Kauppinen, Elizabeth Coulthard

**Affiliations:** a Bristol Medical School, University of Bristol, Bristol, UK; b School of Experimental Psychology, University of Bristol, Bristol, UK; c Clinical Research and Imaging Centre, University of Bristol, Bristol, UK; d Clinical Neurosciences, North Bristol NHS Trust, Bristol, UK

**Keywords:** Prospective memory, Alzheimer's disease, Early marker, Cortical thickness, Magnetic resonance imaging

## Abstract

**Introduction:**

: Prospective memory (PM) is a marker of independent living in Alzheimer's disease. PM requires cue identification (prospective component) and remembering what should happen in response to the cue (retrospective component). We assessed neuroanatomical basis and functional relevance of PM.

**Methods:**

84 older participants (53–94 years old, 58% male) with or without Mild Cognitive Impairment (MCI) performed PM tests, Activities of Daily Living (ADL) scale and had a structural MRI of the brain to estimate for cortical thickness and hippocampal subfield volumes. A General Linear Model cluster analysis was carried out using FreeSurfer to determine which cortical regions were correlated with PM scores.

**Results:**

: Both components of PM are impaired in MCI (*p* < .001). The retrospective component of PM correlates strongly with ADL (*p* = .005). Prospective component performance correlates positively with cortical thickness of bilateral frontal-temporal-parietal cortex and volume of CA1 of hippocampus. In contrast, the retrospective component performance correlates positively with cortical thickness of a right-lateralised fronto-temporal-parietal network and volumes of subiculum and CA3 hippocampal subfields.

**Discussion:**

: Our neuroimaging findings complement and extend previous research into structural correlates of PM. Here, we show that there are distinct, yet, overlapping brain regions correlating with the two components of PM. PM performance provides a window into real-life functional abilities in people at risk of Alzheimer's disease and could be utilised as a marker of clinically relevant disease.

## Highlights

• Event-based prospective memory is impaired in people with mild cognitive impairment.
• Prospective memory mirrors real-world behaviour.
• Structural changes in the brain correlate with prospective memory scores.
• Different aspects of prospective memory correlate with distinct brain networks.
• Prospective memory might be a meaningful measure in clinical practice or research.

## 1 Introduction

*Prospective Memory* (PM) is remembering to perform an action at the appropriate time. PM can be event-based, such as remembering to buy a bottle of milk on the way back from work, or time-based, such as remembering to turn the hob off in 20 min time (Einstein et al., 1992). In contrast to retrospective memory, which deals with remembering events that happened in the past, PM deals with actions to be undertaken in the future. Given its reliance on self-initiated retrieval, PM is one of the most cognitively demanding processes (Craik, 1986).

PM can be further divided into two principal components – prospective and retrospective. The prospective component is the ability to recognise the appropriate cue at which the action is to be performed, also called “cue identification”. The retrospective component is remembering what the action to be performed is, also called “intention retrieval” (McDaniel and Einstein, 1992).

Impaired PM may be an important determinant of ability to perform day-to-day activities in people destined to develop Alzheimer's disease (AD) dementia (Burgess et al., 2000; Kliegel and Martin, 2010). 50–80% of all reported everyday memory problems consist of some form of PM impairment (Terry, 1988) and around 40% of patients visiting memory clinics have problems in PM (Kliegel and Martin, 2010). PM may be a *functionally relevant* marker of AD. Such a marker would be complementary to biological measures (such as amyloid presence on PET) that demonstrate the presence of pathology rather than its effect on day-to-day performance (Bergeron et al., 2018).

Tests of episodic memory, such as delayed verbal recall, are the most widely used and sensitive cognitive tests for early AD (Albert, 1996; Locascio et al., 1995). There are similarities between episodic memory and PM. Indeed, at least the retrospective component of PM partially depends on intact episodic memory (Martin et al., 2007). However, given the complex and cognitively taxing nature of PM, it might be susceptible to the effects of AD earlier in the course of the illness than episodic memory. This supports the theory that impaired PM is an early marker of the effects of AD (e.g. Huppert and Beardsall, 1993; Huppert et al., 2000; Maylor et al., 2002).

When considered as a whole, successful PM requires intact networks that include prefrontal cortical regions, particularly rostral prefrontal cortex (rPFC) (also referred to as anterior prefrontal cortex or BA10), as well as medial temporal lobe and the parietal cortex (Burgess et al., 2002, 2001, 2003; den Ouden et al., 2005; Martin et al., 2007; Okuda et al., 1998; Palmer and McDonald, 2000; Shallice and Burgess, 1991). Widespread brain involvement is no surprise considering the demands and multicomponent nature of PM. Prospective and retrospective components of PM are very likely to require different brain networks (Simons et al., 2006). In patients affected by AD, these networks may be differentially affected by disease and amenable to distinct forms of therapy.

Here, we explored the neurobiological basis and relevance to day-to-day life of distinct components of PM in a cohort of older people with a spectrum of memory disorders. We have further divided the prospective memory scores into the two components (prospective and retrospective) of PM in order to better understand which aspects of PM are particularly important for day-to-day life. To infer which brain regions may relate to PM performance in our cohort, cortical thickness measures were used to examine changes in brain structure. Reduction in cortical thickness is a highly reproducible metric (Govindarajan et al., 2014; Han et al., 2006; Wonderlick et al., 2009) and is linked with progression to neurodegenerative diseases such as AD (Clarkson et al., 2011; Hartikainen et al., 2012), more precisely than other structural MRI metrics. We also extracted hippocampal subfield and entorhinal cortex volumes using the Automated Segmentation of Hippocampal Subfields (ASHS) software to obtain highly detailed information about structure of medial temporal lobe, as this region is critical for episodic memory generally.

Using these techniques, we tested the hypotheses that PM is impaired in Mild Cognitive Impairment (MCI) and this relates to difficulty with everyday tasks. Furthermore, we explored how variation of regional cortical thickness and hippocampal volume between individuals predicts PM performance.

## 2 Methods

### 2.1 Participant population

84 participants with an age range of 53 to 94 were recruited from a variety of registered databases such as the Join Dementia Research database, local GPs, the Memory Clinic at the North Bristol NHS Trust and the Avon and Wiltshire Mental Health Partnership NHS Trust's Everyone Included database. Participants were classified into the following 3 groups: Controls, Subjective Cognitive Decline (SCD) and MCI using an algorithm presented in **Supplementary Fig. 1**. In brief, standard diagnostic criteria were used to diagnose MCI clinically (Albert et al., 2011), participants without MCI were classified as SCD rather than healthy controls if they responded ‘Yes’ to 2 or more of the questions shown in **Supplementary Table 1** and scored above or equal to 88 on Addenbrooke's Cognitive Examination-III (ACE-III) (Crawford et al., 2012) and received less than or equal to 0.5 on Clinical Dementia Rating Scale (CDR) (Morris, 1993).

### 2.2 Standard protocol approvals and participant consents

This study has been reviewed and approved by the NHS Frenchay Research Ethics Committee. Participants provided written informed consent to take part in this study prior to any form of testing.

### 2.3 Data collection

Demographic data (e.g. age, sex, Crawford's IQ (Crawford and Allan, 1997), education), medical history and current medication use was obtained from participants during the initial telephone screening.

#### 2.3.1 Cognitive and neuropsychological tests

Participants were assessed using Clinical Dementia Rating (CDR; to categorise severity of cognitive symptoms/dementia) and Addenbrookes Cognitive Examination-III (ACE-III; to assess attention, memory, fluency, language and visuospatial abilities) – the information obtained from these tests was used in participant classification (see above). Participants’ day-to-day quality of living was assessed using the 22-item Nottingham Extended Activities of Daily Living Scale (ADL) self-report questionnaire (Nicholl et al., 2002). The 22-item scale is scored on the basis of the frequency of performing each activity. The overall ADL score is a collection of scores obtained from four generic categories of daily living: Mobility (Q1-6), Kitchen (Q7-11), Domestic (Q12-16) and Leisure (Q17-22). A score of 0 was given if the participant states that they perform an activity “never” or “with help”. A score of 1 was given if the participant has chosen “on my own with difficulty” or “on my own”. The maximum score is 22, where a higher score represents a higher level of independence in day-to-day functioning.

Event-based PM assessment was carried out using the Rivermead Behavioural Memory Tests – Third Edition (RBMT-3) (Wilson et al., 1989). RBMT-3 is a battery of 10 memory tests with high ecological validity. For the purposes of this study, only the data from the 3 PM tests (Appointments, Messages and Belongings) were analysed and reported. Our distinct addition to this protocol was in the scoring of the tasks to divide into prospective and retrospective components.

##### 2.3.1.1 Prospective memory subcomponent scoring

In this study, the same scoring method as Huppert and Beardsall(1993) was used and a new protocol for the assessment of the prospective and retrospective components of PM was developed. Descriptions of subtest and the protocol for scoring the prospective component and retrospective component are provided below.

*“Appointments*”: An alarm is set for 25 min after the start of the testing session. The experimenter says:

“When the alarm rings you have to ask me two questions. The questions are - (1) When does this session end? and (2) when will I know the results of this test?”. Testing session then commences. After 25 min, the alarm sounds and the experimenter waits for the participant to respond. If no response after 10 seconds, the experimenter prompts with: “Do you remember what you were going to do when the alarm rang”.

*“Belongings”:* The experimenter asks to borrow two belongings (e.g. a key ring) of the participant. Then, the belongings are placed in two distinct locations while the participant is watching. The experiment says:

“Please ask for both of your belongings and tell me where they are, when I say, ‘This is the end of the test.’”.

The testing session continues, and when the cue is given the experimenter waits for the participant to respond. If no response is forthcoming after 10 seconds, the experimenter prompts with: “Do you remember what you were going to do at the end of the testing session”.

*“Messages” (immediate and delayed recall):* This task is carried out as a part of the *“Route”* task where the participant is asked to retrace a 6-point route around the room shown by the examiner. The experimenter first demonstrates the route to the participant:

Before demonstrating the route, the experiment picks up two items (a book and an envelope), which are placed in two distinct locations during the 6-point route. When the participant is performing the task, if they forget to pick up the items, the experimenter prompts them to ensure they do so, since they will also be assessed on remembering the correct location for each item.

This task is carried out twice, once as an immediate recall task and then, at a later time, as a delayed recall task (without demonstration from the examiner).

A descriptive example of how one of the tasks was scored: “Appointments”

*Prospective component:* 1 point for spontaneously asking each question i.e. without prompt (regardless of accuracy of content of questions). No points were given if any prompting was required. Max raw score: 2.

*Retrospective component:* 1 point for each question correctly remembered. Max raw score: 2.

Please see Table 1 for a detailed breakdown of scores for each prospective memory task.

**Table 1:** Detailed breakdown of scoring system for each of the 3 prospective memory tasks from RBMT-3.

Prospective Memory Task	PM subcomponent	Maximum possible score for Items		Maximum possible score for Locations	
		Item 1	Item 2	Location 1	Location 2
**Appointments**	Prospective (cue identification)	1	1	N/a	N/a
	Retrospective (intention retrieval)	1	1	N/a	N/a
**Belongings**	Prospective (cue identification)	1	1	1	1
	Retrospective (intention retrieval)	1	1	1	1
**Messages (Immediate)**	Prospective (cue identification)	1	1	N/a	N/a
	Retrospective (intention retrieval)	1	1	1	1
**Messages (Delayed)**	Prospective (cue identification)	1	1	N/a	N/a
	Retrospective (intention retrieval)	1	1	1	1
Total score	24				

**NOTE.** Participants received points for the ‘prospective/cue-identification' component of PM if they spontaneously carried out the task (i.e. without being prompted), if they required a prompt, they did not receive any scores for this subcomponent, max raw score 10. Participants received points for the ‘retrospective/intention-retrieval' component of PM for correctly remembering what they needed to do (max raw score 14).

The total PM raw score was calculated as the sum of the raw scores from each of 3 subtests. The total PM score was then converted into a percentage of maximum available raw score for all tasks (i.e. 24).

#### 2.3.2 Magnetic resonance imaging procedures

##### 2.3.2.1 Image acquisition

All MRI scans were undertaken using a Siemens Magnetom Skyra 3T system. The system was also equipped with a 32-channel head receiver array coil and a parallel transmit body coil. The imaging protocol used in this study was as follows:

*3D T1-weighted Magnetisation-Prepared Rapid Gradient Echo (MPRAGE) with the parameters*: sagittal, TR 2200 ms, TE 2.28 ms, TI 900 ms, flip angle 9°, FOV 220 × 220 × 179 mm, acquired resolution 0.86 × 0.86 × 0.86 mm, acquired matrix size 256 × 256 × 208, acquisition time 5 min and 7s. *Multi-contrast TSE with the parameters:* Coronal, TR 7500ms, number of echoes: 3, TE 9.1, 72 & 136 ms, acquired resolution 0.69 × 0.69 × 1.5 mm, reconstructed resolution 0.34 × 0.34 × 1.5 mm (after 2-fold interpolation in-plane by zero-filling in k-space, and inclusive of 15% slice gap), GRAPPA factor 2, FOV 220 × 220 × 34, acquired matrix size 270 × 320 × 58, acquisition time 5-min and 9s. Note: this scan was not ‘whole-brain’, it’s coverage only extending approx. 1cm beyond anterior and posterior ends of the hippocampus. These scans were tilted such that the hippocampal body lay perpendicular to the slice acquisition plane.

##### 2.3.2.2 Image processing

Images were processed using *FreeSurfer* software version 6.0 (https://surfer.nmr.mgh.harvard.edu) (Fischl et al., 2001). Further detailed information regarding the procedures of image analysis done using *FreeSurfer* can be found in Fischl et al. (2001). In brief, the semi-automated pipeline includes processes such as normalisation of intensity, skull stripping, cerebral white matter segmentation as well as the estimation of the grey/white matter boundary (Dale et al., 1999). Following topological defect corrections, the grey/white matter boundary is used to locate the pial surface and cortical thickness (shortest direct distance between the white matter surface and pial surface) was then measured (Fischl and Dale, 2000). This method has been validated (Rosas et al., 2002) and it has been shown to be reliable (Dickerson et al., 2008; Han et al., 2006). Hippocampal subfields (CA1, CA2, CA3, dentate gyrus and subiculum) and entorhinal cortex were demarcated using the automated hippocampal subfield segmentation (ASHS) software (rev103, 12/06/2014) (Yushkevich et al., 2015), using the UPENN atlas consisting of scans of MCI patients and older adults (dated 16/04/2014).

### 2.4 Statistical analyses

#### 2.4.1 Behavioural data analysis

All behavioural data analysis was undertaken using IBM SPSS version 24. GraphPad Prism version 7 and Microsoft Office software (Excel) were used for visualisation of behavioural analysis results (e.g. graphs, tables).

A Pearson's chi-squared test was run to check for gender balance within groups. A univariate analysis of variance (ANOVA) was performed to check whether there was an age difference between groups. Since the data violated assumptions of normality and age was identified as a covariate, the non-parametric analysis of covariance test (Quade's test) was performed with post hoc pairwise comparisons to compare PM performance across groups and the Spearman partial correlation test (age as covariate) was run to investigate the relationship between PM performance and ADL.

In all analyses, *p* < .05 was accepted as statistically significant. In instances where multiple comparisons were undertaken, a Bonferroni correction for multiple tests was used.

#### 2.4.2 Cluster analysis

*FreeSurfer* was used to carry out vertex-wise statistical analyses using a General Linear Model with a “Different Onset, Different Slope” design. Initially, participant-specific cortical surface data was registered to an average “study-specific” template. The volume and thickness values were smoothed with a full width at a half maximum (fwhm) value of 10 mm. Age was accounted for as a covariate in this model and a Montecarlo multiple comparisons correction was done with a .05 threshold for significance (Hagler et al., 2006).

Group-based correlations were not carried out, due to a lack of statistical power given individual group sample sizes.

#### 2.4.3 Hippocampal volume analysis

T1-weighted MPRAGE (typically not brain-extracted), and the echo-summed T2-weighted image from the multi-echo sequences (brain extracted using the FSL programme ‘bet2’) were used as input to ASHS. All hippocampal masks created as an output of ASHS were visually inspected for quality. In cases where the multi-echo image was either not present or of too poor quality due to movement artefacts, the single echo TSE was used instead. We have shown in-house that ASHS outputs from either scan-type are not significantly different from one another. Volumes were all normalised to intracranial volume.

A partial correlation analysis, controlling for age, was performed to investigate whether volumes of hippocampal subfields and entorhinal cortex were significantly correlated with the prospective and retrospective components of PM. A *p-*value lower than .01 was accepted as statistically significant (to Bonferroni correct for multiple comparisons).

## 3 Results

### 3.1 Participant demographics

A total of 84 participants took part in the study (demographic information in Table 2). All 84 participants took part in the behavioural measurement of PM using RBMT-3. 71 participants completed both RBMT-3 and ADL and, after exclusions due to contraindications, 59 participants underwent an MRI scan.

**Table 2:** Summary of the demographic data of all 3 participant groups.

Demographic Variable	HC	SCD	MCI	*p*value
***N***	26	29	29	
**Age (years)**	71.40 ± 7.51	71.10 ± 8.14	78.59 ± 9.16	.068
**Age range**	61–89	55–86	53–94	
**Sex (male)**	46.15%	58.62%	68.96%	.230
**IQ**	108.91 ± 11.08	107.68 ± 9.14	104.00 ± 9.14	.101
**Handedness (% right-handed)**	88%	82%	93%	.512
**ACE-III (Total)**	95.55 ± 2.37	93.85 ± 3.73	79.47 ± 8.37	<.001
**ACE-III: ATTENTION (/18)**	16.15 ± 2.78	17.28 ± 1.02	16.29 ± 2.31	<.001
**ACE-III: MEMORY (/26)**	21.50 ± 6.18	22.24 ± 3.67	21.29 ± 4.05	<.001
**ACE-III: FLUENCY (/14)**	10.75 ± 3.01	11.80 ± 1.63	10.33 ± 2.29	<.001
**ACE-III: LANGUAGE (/26)**	24.30 ± 1.78	24.68 ± 2.48	24.14 ± 1.85	=.001
**ACE-III: VISUOSPATIAL (/16)**	15.05 ± 1.78	15.36 ± 1.11	15.19 ± 1.17	=.001

**NOTE.** Results across groups represent mean ± SD, except ranges and percentages. There were no significant differences between groups in age, gender, or IQ, but the MCI group trended towards being slightly older and analyses throughout the remainder of the manuscript control for age. ACE-III (total) was significantly different across groups. Pairwise comparisons showed that ACE-III (total) score (as well as all ACE-III sub-scores) of MCI group was significantly lower than both HC and SCD groups (*p <* .001). However, there was no significant difference between the ACE-III (total) score (or ACE-III sub-scores) of HC and SCD groups (*p* = .925).

### 3.2 Behavioural results

#### 3.2.1 Prospective memory is impaired in people with Mild Cognitive Impairment

People with MCI performed significantly worse in total PM (F(2, 81) = 39.14, *p* < .001) as well as in both the prospective and retrospective components of PM (F(2, 81) = 33.98, *p* < .001 and F(2, 81) = 21.14, *p* < .001, respectively), whilst controlling for age, in keeping with our hypothesis. There was no significant difference in total PM performance between controls and people with SCD (*p* = .078). This was also true for the retrospective (*p* = .777) and prospective (*p* = .180) components. Raw scores for the prospective and retrospective components of each PM task per group are provided in Table 3.

**Table 3:** Average raw scores for each PM task per group.

Prospective memory task	PM subcomponent	Participant group	Average raw score (Mean ± SD)	Percentage correct raw score (%)
**Appointments**	Prospective (/2)	HC	1.19 ± 0.98	59.92
		SCD	1.25 ± 0.89	62.50
		MCI	0.72 ± 0.96	36.21
	Retrospective (/2)	HC	1.14 ± 0.85	57.14
		SCD	1.29 ± 0.85	64.29
		MCI	1.29 ± 0.85	64.29
**Belongings**	Prospective (/4)	HC	1.57 ± 1.36	39.29
		SCD	1.75 ± 1.21	43.75
		MCI	1.38 ± 1.35	34.48
	Retrospective (/4)	HC	3.24 ± 1.41	80.95
		SCD	3.79 ± 0.69	94.64
		MCI	3.38 ± 1.15	84.48
**Messages (Immediate)**	Prospective (/2)	HC	1.76 ± 0.62	88.10
		SCD	1.71 ± 71	85.71
		MCI	1.86 ± 0.52	93.10
	Retrospective (/4)	HC	3.38 ± 1.16	84.52
		SCD	3.36 ± 1.06	83.93
		MCI	3.31 ± 0.76	82.76
**Messages (Delayed)**	Prospective (/2)	HC	1.57 ± 0.81	78.57
		SCD	1.57 ± 1.00	78.57
		MCI	1.31 ± 0.97	65.52
	Retrospective (/4)	HC	3.14 ± 1.39	78.57
		SCD	3.46 ± 1.14	86.61
		MCI	3.03 ± 1.09	75.86

**NOTE.** Average raw scores with SD and percentage correct raw scores for the prospective and retrospective components of the three prospective memory tasks: appointments, belongings and messages, for each participant group: HC, SCD and MCI.

### 3.3 Retrospective component of Prospective Memory is related to performance in everyday life

Total PM performance correlated significantly with scores in the Activities of Daily Living Questionnaire, whilst controlling for age, in keeping with the predicted role of PM in maintaining day-to-day independence (*N* = 71, *p < .05). Only the retrospective component of PM and not the prospective component were correlated with Activities of Daily living Score (Table 4). As a comparison, ACE-III score also significantly correlated with Activities of Daily Living Score (partial correlation *r* = .335, *p* = .006).

**Table 4:** Partial correlations between Activities of daily living and Prospective Memory.

Variables	Partial correlation *r*	*p*value
Total PM vs ADL	0.255*	.033*
Pros. Comp. vs ADL	0.201	.095
Retro. Comp. vs ADL	0.329*	.005*

†**NOTE.** Results of the partial correlation analysis investigating the relationship between (i) Total Prospective Memory, (ii) Prospective Component of Prospective Memory (Pros. Comp.), (iii) Retrospective Component of Prospective Memory (Retro. Comp.) and Nottingham Extended Activities of Daily Living scores (ADL). (*N* = 71)

**Abbreviations:** PM, prospective memory; ADL, activities of daily living; Pros. Comp., prospective component; Retro. Comp., retrospective component.

### 3.4 Brain volume relationships with Prospective Memory

Cluster analysis of cortical thickness of brain regions associated with event-based PM revealed a network including temporal lobe (middle temporal and fusiform regions), frontal lobe (caudal middle frontal, superior frontal regions e.g. rostral prefrontal cortex) and parietal lobe (inferior parietal, supramarginal and isthmus cingulate regions) (see Fig. 2). Within the hippocampus, CA1 volume was highly positively correlated with PM.

When exploring the two components of PM separately, the prospective component (i.e. cue-identification) positively correlated with cortical thickness of frontal lobe (superior frontal, caudal middle frontal regions), parietal lobe (precuneus, supramarginal, isthmus cingulate regions), temporal lobe (inferior temporal, superior temporal and fusiform regions) and occipital lobe (lateral occipital region). In hippocampal regions, bilateral CA1 and right-lateralised entorhinal cortex volumes were significantly positively correlated with the prospective component of PM (see Fig. 3).

In contrast, retrospective component (i.e. intention retrieval) involves a different, more lateralised (right-hemisphere) network (see Fig. 3). In the left hemisphere, only the cortical thickness of fusiform region in the temporal lobe was significantly positively correlated with the retrospective component. However, in the right hemisphere, there was a larger network of positive correlations between the retrospective component and cortical thickness of frontal lobe (superior frontal and medial orbitofrontal regions), parietal lobe (superior parietal and isthmus cingulate regions) and temporal lobe (superior temporal and parahippocampal regions) as well as the CA1 (positive) and CA3 (negative) hippocampal subfield volumes (see Table 5).

**Table 5:** Hippocampal volume correlations with the two components of Prospective Memory.

PM component	CA1	CA3	Subiculum	Entorhinal cortex	Total hippocampal volume
**Pros. Comp. (Left)**	0.424^⁎⁎^	−0.103	0.268	0.221	0.296
**Pros. Comp. (Right)**	0.385^⁎⁎^	−0.161	0.250	0.418^⁎⁎^	0.264
**Retro. Comp. (Left)**	0.296	−0.168	0.398	0.227	0.213
**Retro. Comp. (Right)**	0.327*	−0.422^⁎⁎^	0.256	0.268	0.177

‡**NOTE.** Data shows ‘partial r’ from hippocampal subfield and entorhinal cortex volume correlation analysis between the prospective (Pros. Comp.) and retrospective (Retro. Comp.) components of PM across the left and right hemispheres, respectively. (*=<.01, ^⁎⁎^=<.005, to account for multiple comparisons)

**Prospective Component left hemisphere** significantly positively correlated with CA1 volume only.

**Prospective Component right hemisphere** significantly positively correlated with CA1 and Entorhinal Cortex volumes.

**Retrospective Component left hemisphere** did not significantly correlate with any hippocampal region.

**Retrospective Component right hemisphere** significantly positively correlated with CA1 and negatively correlated with CA3 volumes.

**Abbreviations:** PM, prospective memory; Pros. Comp., prospective component; Retro. Comp., retrospective component.

## 4 Discussion

PM is impaired in MCI and the retrospective component of PM strongly correlates with ability to perform activities of daily life. Brain structure in two overlapping, but distinct, brain networks correlates with performance in the retrospective and prospective components of PM. The prospective component is associated with bilateral prefrontal, temporal, and parietal cortical thickness and medial temporal lobe volume (particularly CA1 subfield of hippocampus). In contrast, the retrospective component performance correlates with unilateral (right-hemisphere) medial temporal lobe volume (particularly with CA1 subfield and inversely with CA3 subfield) and thickness of a right-lateralised fronto-temporal-parietal cortical network.

**Fig. 1:**
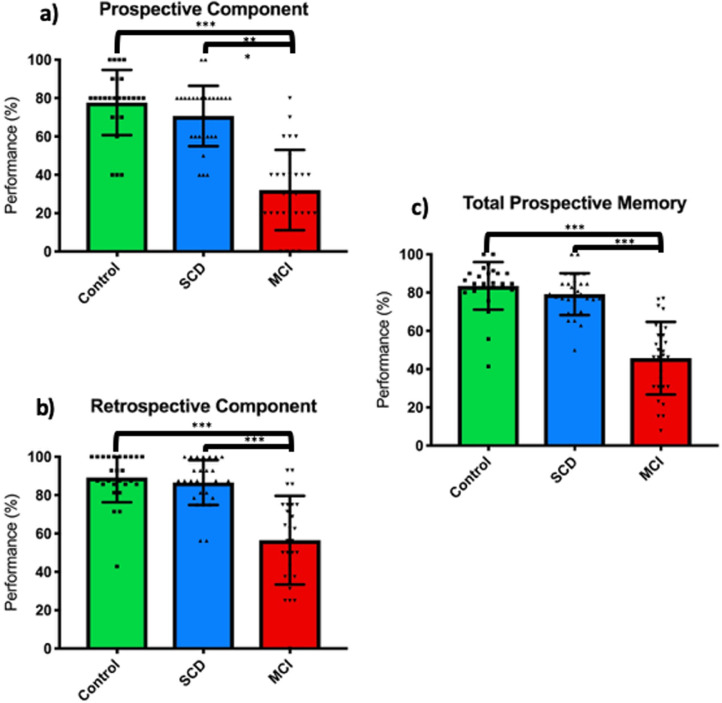
Prospective Memory in three participant groups: Healthy Controls, Subjective Cognitive Decline and Mild Cognitive Impairment. Performance of the 3 groups (HC (*N* = 26), SCD (*N* = 29) and MCI (*N* = 29)) presented as total PM score (%). People with MCI performed significantly worse than people with SCD or HC in both the prospective component and retrospective components of Prospective Memory tasks (^⁎⁎⁎^=*p* < .001) (*N* = 84). Error bars represent SD. **Abbreviations:** SCD, subjective cognitive decline; MCI, mild cognitive impairment.

The behavioural distinctions between healthy participants and those with MCI are in line with previous findings in this field (e.g. McDaniel et al., 2013, 2015). A possible theoretical explanation for this could be that cognitive processes involved in intention retrieval (e.g. attentional processes) might be impaired in very early stages of AD (e.g. MCI) (McDaniel et al., 2013; Shelton et al., 2011). This theory is further supported by neuroimaging studies demonstrating medial temporal lobe regions (e.g. hippocampus) are important for associative retrieval in PM and these structures are amongst the first to deteriorate in very early AD (Buckner, 2004; Jack et al., 2008; Moscovitch, 1994). Further theoretical explanations for PM failure in patients with MCI is suggested to be due to deficits in strategic monitoring (a crucial process for cue-identification) in people at very early stages of AD (including those with MCI) (Blanco-Campal et al., 2009; Castel et al., 2009). This theoretical explanation is concordant with the poor performance of the MCI group in the prospective component of PM (<30% accuracy) shown in Fig. 1.

**Fig. 2:**
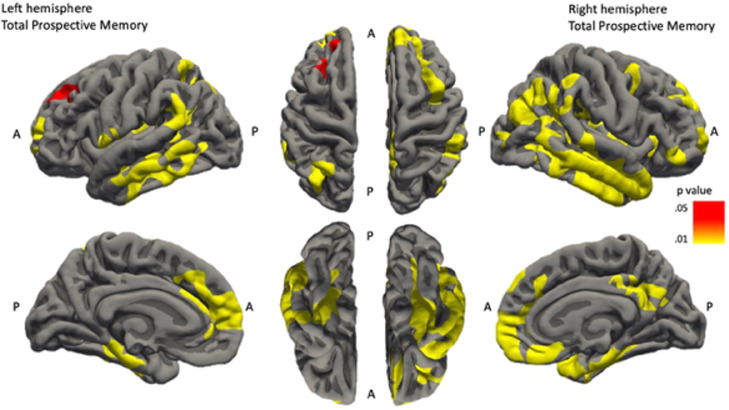
Brain maps demonstrating regions where changes in cortical thickness are significantly positively correlated with Total Prospective Memory scores. In all brain maps; top row demonstrates lateral and superior views of the brain and the bottom row represents the medial and inferior views of the brain. **Total Prospective Memory left hemisphere** 4 correlation clusters: middle temporal (cluster-wise *p* value = .0002), superior frontal (2 clusters - cluster-wise *p* values = .0002 and  = .0402), supramarginal (2 clusters - cluster-wise *p* values = .0002 and  = .0058), inferior parietal (cluster-wise *p* value = .0002) (*N* = 59). **Total Prospective Memory right hemisphere** 4 correlation clusters: fusiform (cluster-wise *p* value = .0002), superior frontal (cluster-wise *p* value = .0002), caudal middle frontal (cluster-wise *p* value = .0002) and isthmus cingulate (cluster-wise *p* value = .0054) (*N* = 59).

**Fig. 3:**
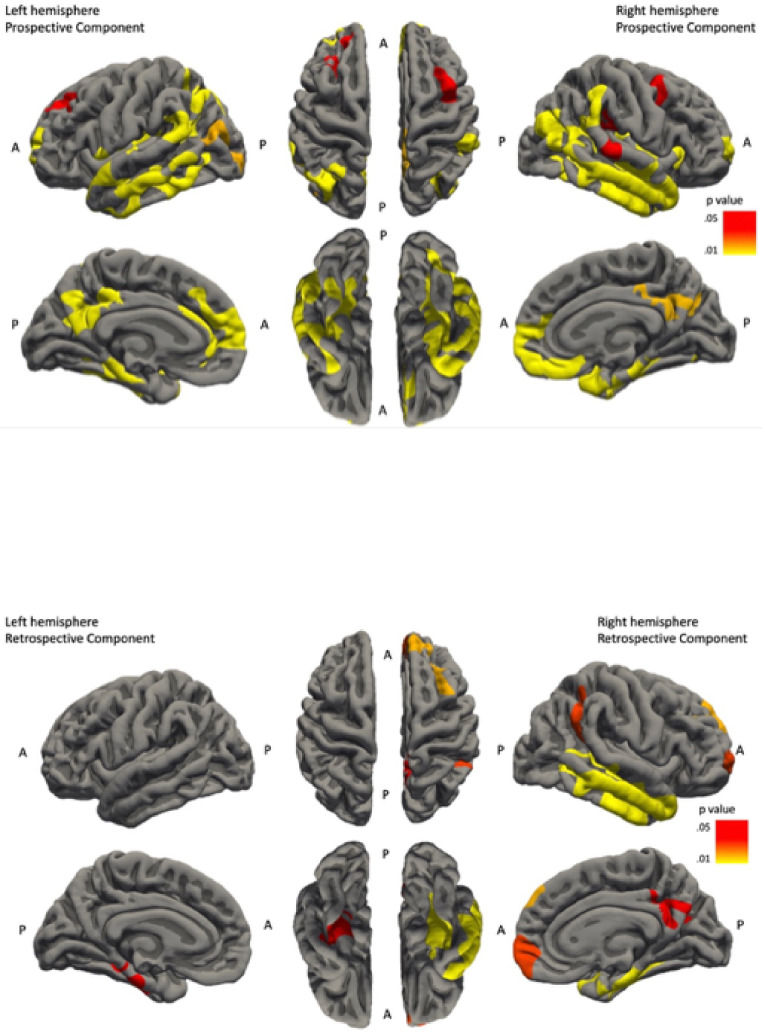
Brain maps demonstrating regions where changes in cortical thickness are significantly positively correlated with Prospective Component and Retrospective Component scores. **Prospective Component left hemisphere** 7 positive correlation clusters: inferior temporal (cluster-wise *p* value = .0002), superior frontal (cluster-wise *p* value = .0002), inferior parietal (cluster-wise *p* value = .0002), precuneus (cluster-wise *p* value = .0002), supramarginal (cluster-wise *p* value = .0038), lateral occipital (cluster-wise *p* value = .0124) and superior frontal (cluster-wise *p* value = .0276) (*N = 59)*. **Prospective Component right hemisphere** 5 positive correlation clusters: fusiform (cluster-wise *p* value = .0002), superior frontal (cluster-wise *p* value = .0002), isthmus cingulate (cluster-wise *p* value = .01196), superior temporal (cluster-wise *p* value = .0229) and caudal middle frontal (cluster-wise *p* value = .0441) (*N = 59)*. **Retrospective Component left hemisphere** 1 positive correlation clusters: fusiform (cluster-wise *p* value = .02997) (*N* = 59). **Retrospective Component right hemisphere** 6 positive correlation clusters: superior temporal (cluster-wise *p* value = .0002), parahippocampal (cluster-wise *p* value = .0006), superior frontal (cluster-wise *p* value = .0124), medial orbitofrontal (cluster-wise *p* value = .0179), superior parietal (cluster-wise *p* value = .0181) and isthmus cingulate (cluster-wise *p* value = .0252) (*N* = 59).

PM performance was similar between people with SCD and controls with the only abnormality being in those with MCI (Bolló-Gasol et al., 2014; Karantzoulis et al., 2009; Kazui et al., 2005; Troyer and Murphy, 2007). This questions how useful PM would be as a very early marker of incipient dementia, if abnormalities are only detectable at the MCI phase when we know that performance on standard neuropsychological tests will differ between MCI and healthy controls. Previous studies have shown a defect in PM in people with SCD (Hsu et al., 2015). There are several reasons why we may not have shown this here. First, SCD is a heterogenous group with a high degree of uncertainty in the number of people who will go on to develop MCI and AD over coming years (Archer et al., 2015). We do not know which participants in our cohort are going to develop AD, but we do know that there may be a high number in both the healthy control and SCD groups with abnormal amyloid profiles and a high risk of developing AD (Schott et al., 2010). So, impaired PM may be an early marker of AD and yet not differ between seemingly healthy participants and those with SCD. Furthermore, different ways of testing PM may be more sensitive to early AD – more naturalistic tasks may be more sensitive and are worthy of future investigations (Lee et al., 2018).

We are not arguing that PM will be the earliest marker of AD – we already know that amyloid status can detect AD many years before diagnosis of dementia. Instead, we propose that PM testing should be an important part of the suite of tests used to determine the functional effects of accruing AD pathology. While we routinely now use lumbar puncture in clinical practice to detect the levels of amyloid and tau in cerebrospinal fluid (National Institute for Health and Care Excellence (UK), 2018), prognostication for patients would be improved if we could determine the impact of abnormal biology on day-to-day life. We know that factors, such as frailty, mediate the relationship between AD pathology and the clinical presentation of dementia (Wallace et al., 2019). We show here that PM might help to determine who has manifest susceptibility to the effects of AD and, therefore, could be targeted with disease modifying or supportive therapy and clinical trials.

Only the retrospective component of PM significantly correlates with functional independence in daily life. It is possible that people make use of strategies to compensate for loss of prospective component – such as telephone reminders – so the prospective component of PM is less detrimental to ADL. Thus, when the retrospective component is also impaired, an impact on day-to-day functioning becomes obvious. Unsurprisingly, ACE-III performance also correlated with Activities of Daily Living. ACE-III probes a broad range of cognitive processes, including retrospective memory and may be a sensitive but non-specific marker of impairment.

We were somewhat surprised that it was the retrospective and not prospective component of PM that most closely correlated with functional ability. As our scoring system was a post hoc bolt-on to an existing test, we considered whether it could bias towards sensitivity to the retrospective component. One possibility is that prompting prospective component triggered the retrospective component. However, if this were the case, we would expect the retrospective component to be a less rather than more sensitive measure. Further testing, perhaps with a different PM protocol is required to validate our findings.

Neuroimaging (e.g. structural or functional MRI, PET, CT) can tell us a lot about the changes occurring in the brain due to AD (e.g. hippocampal atrophy). These changes have the potential to be particularly important for clinical practice and trials if they can act as a proxy measure of functional performance – increasing precision and reducing noise of outcome measures. In this study, we demonstrated that distinct sets of networks are involved in the two components of PM. Our findings are in line with the literature on the role of rostral prefrontal cortex in PM (Burgess et al., 2001, 2003; Okuda et al., 1998; Simons et al., 2006).

In addition, our findings are in concordance with the theoretical basis of the prospective and retrospective components of PM. The distinct, but overlapping, brain regions involved in the two subcomponents of PM reflect the complex cognitive and neural basis of the two processes: cue identification (prospective component) and intention retrieval (retrospective component) (Burgess et al., 2011, 2003; Ellis et al., 1999). In an event-based PM task (such as the ones reported in this study), the prospective component requires cognitive processes involved in self-initiation and recognition of a cue, whilst undertaking an “ongoing task” (to fill in the time period from generating the intention until the appropriate event to act upon occurs), during a period known as the “retention interval” (Burgess et al., 2003). The role of the “ongoing task” is to prevent participants from continuously rehearsing the intention, making this a prospective memory, instead of a working memory task.

On the other hand, the theoretical basis of the retrospective component of PM is harder to distinguish from other cognitive processes such as “retrospective memory”. One highly plausible explanation for this could be that PM partly depends on processes involved in retrospective memory (i.e. the retrospective component of PM). This has also been supported by neuroimaging studies demonstrating an overlap of brain regions involved in PM (e.g. rPFC) and retrospective memory (Grady, 1999). Although these two types of memory have theoretical differences, e.g. encoding of information in retrospective memory could be either incidental or intentional, whereas due to the nature of PM, encoding needs to be intentional, they appear to share similar underlying neural processes that support retrieval of information (West and Krompinger, 2005).

Structural correlates presented here extend previous functional imaging work that demonstrate intention retrieval (retrospective component of PM) is associated with increased blood flow to lateral prefrontal cortex, posterior cingulate and precuneus and parietal cortex, whereas, cue identification was selectively associated with anterior cingulate (Burgess et al., 2001, 2003; den Ouden et al., 2005; McDaniel et al., 2013; Okuda et al., 1998; Simons et al., 2006). The difference between our structural and previous functional imaging findings may reflect inherent differences in the two measures. Structural correlations with behavioural performance in our participant group reflects the way in which acquired brain changes affect PM performance. While this may give some insight into brain areas normally involved in PM, one cannot make direct inferences about the normal brain networks involved in PM as they may, for example, not vary significantly with disease and, therefore, would not necessarily correlate with behaviour. In contrast, functional imaging shows the areas in normal brain where blood flow increases during PM. Nevertheless, there is significant overlap between the structural changes that correlate with PM in our study and those found to be associated with PM in previous studies using fMRI (Simons et al., 2006).

Structural brain changes associated with functionally relevant aspects of PM open up the possibility of using imaging as a proxy marker for PM in clinical trials of older adults at risk of dementia. Imaging outcome measures avoid confounds of behavioural testing and may allow smaller numbers of patients in clinical trials. If imaging markers can be unequivocally linked to real-life functional ability, then their utility increases.

An unexpected finding was that the CA3 hippocampal subfield negatively correlated with retrospective component of PM. While, this could be an anomaly, it could also have a biologically plausible explanation. CA3 has a well-defined auto-associative structure important for pattern completion and object identification that is possibly relatively less affected by age than other hippocampal subfields (Dillon et al., 2017). If CA3 acts to reinforce similarity between remembered events and objects, it could plausibly impair recalling distinct events at defined times, thus, worsening PM performance.

Our findings are consistent with PET and fMRI findings, showing the anterior prefrontal cortex (BA10; rPFC), an area suggested to be a fundamental part of the network supporting attentional processing (a crucial aspect for cue identification (prospective component of PM)), is involved in PM (Burgess et al., 2011, 2001; Reynolds et al., 2009; Simons et al., 2006). The outcomes of this study extend previous findings regarding the involvement of hippocampus in the information retrieval phase of PM (i.e. retrospective component of PM) (Gordon et al., 2011), by demonstrating which specific hippocampal subfields are involved in this process. This is also in line with the theoretical basis of hippocampus, in particular its role in recognition memory (Eichenbaum et al., 1994, 2007). It is suggested that successful PM performance depends on recognition memory due to the associative nature of PM (e.g. when generating an intention, a link between the cue and intended action is created) (Gordon et al., 2011; Moscovitch, 1994). The importance of hippocampus for memory is thought to be due to its unique relational nature, allowing the formation and retrieval of associative memories (Konkel and Cohen, 2009; Moscovitch, 1994). Although our findings are in line with these theories, it is important to note that findings from a structural neuroimaging study might not directly map onto cognitive processes. Therefore, we suggest that the involvement of hippocampal subfields during the retrieval phase of PM is further investigated using functional neuroimaging methods.

In conclusion, PM performance is shown to be a strong indicator of day-to-day functioning in this study. Moreover, PM performance was also found to correlate with structural brain changes. Our neuroimaging findings demonstrate considerable cross-method concordance with previous studies using fMRI and suggest structural anatomical changes might reflect day-to-day performance of people at risk of AD (Burgess et al., 2001; Reynolds et al., 2009). On the basis of the strong relationship between PM and Activities of Daily Living demonstrated here, future work should probe the ability of PM to track functional status due to AD over time. It remains an open question as to whether PM will be a more specific predictor of functional decline at certain disease stages than more standard tests of retrospective memory and general cognition. Here, we have used a real-world cohort that spans healthy older people, SCD and MCI. The precise predictive power of PM for AD dementia could be better tested with a deeply phenotyped population where amyloid and tau status are known. Overall, the outcomes of this study provide a valuable insight into the use of PM performance as a guide to functional decline in the very early stages of AD.

## CRediT authorship contribution statement

**Volkan Nurdal:** Conceptualization, Investigation, Data curation, Formal analysis, Visualization, Writing - original draft, Writing - review & editing. **Alfie Wearn:** Funding acquisition, Investigation, Data curation, Methodology, Software, Formal analysis, Visualization. **Michael Knight:** Methodology, Software. **Risto Kauppinen:** Methodology, Supervision. **Elizabeth Coulthard:** Methodology, Funding acquisition, Supervision, Writing - review & editing.

## Declaration of Competing Interest

None.

## Appendix Supplementary materials

Image, application 1
