# Supplementary material for: Prospective memory in prodromal Alzheimer's disease: Real world relevance and correlations with cortical thickness and hippocampal subfield volumes
Source: Neuroimage Clin. 2020 Feb 22;26:102226. doi: 10.1016/j.nicl.2020.102226 (PMC7063259; doi:10.1016/j.nicl.2020.102226)
Supplement: Supplementary file 1 [file mmc1.docx]

**Supplementary Figure 1: (colour should be used in print)**


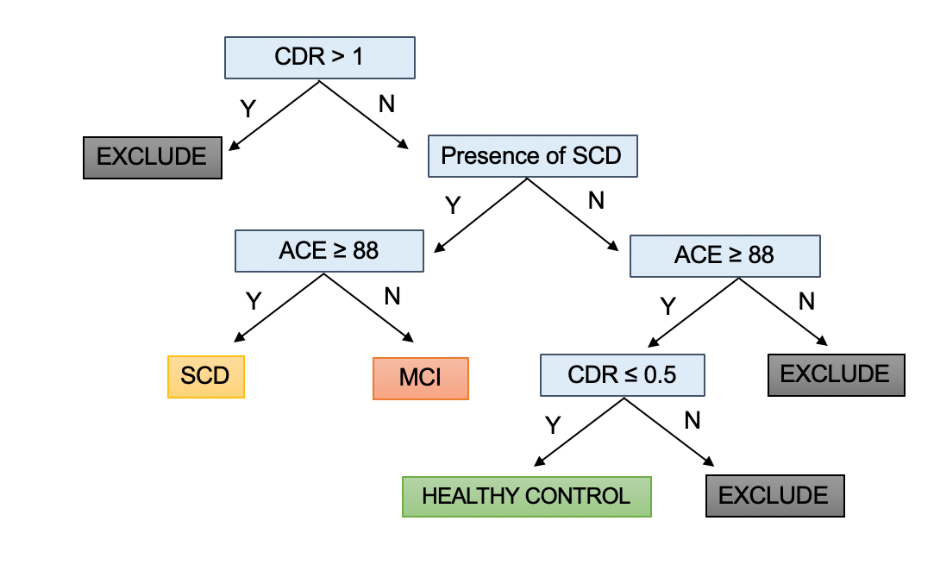


**Figure 1:** Classification criteria used to determine participant groups.

**Abbreviations:** CDR, Clinical Dementia Rating scale; SCD, subjective cognitive decline; ACE, Addenbrooke’s Cognitive Examination Third Edition; MCI, mild cognitive impairment.

**Supplementary Table 1:**

| **Questions** | **Yes / No** |
| --- | --- |
| Are you concerned about your memory? | Y/N |
| Do you think that your memory is worse than 5 years ago? | Y/N |
| Do you think that your memory is poorer than that of other people of a similar age? | Y/N |

**Table 1:** Classification criteria used to determine whether participants will be placed in the SCD group. Participants answering “Yes” to 2 or more of the questions were classified as SCD.
